# Immunization with Epstein–Barr Virus Core Fusion Machinery Envelope Proteins Elicit High Titers of Neutralizing Activities and Protect Humanized Mice from Lethal Dose EBV Challenge

**DOI:** 10.3390/vaccines9030285

**Published:** 2021-03-19

**Authors:** Xinle Cui, Zhouhong Cao, Yuriko Ishikawa, Sara Cui, Ken-Ichi Imadome, Clifford M. Snapper

**Affiliations:** 1 The Institute for Vaccine Research, Department of Pathology, Uniformed Services University of the Health Sciences, Bethesda, MD 20814, USA; zhouhong.cao@nih.gov (Z.C.); scui567@gmail.com (S.C.); clifford.snapper@usuhs.edu (C.M.S.); 2 Center for Cancer Research, National Cancer Institute, Bethesda, MD 20892, USA; 3 Department of Advanced Medicine for Infections, National Center for Child Health and Development, Tokyo 157-0074, Japan; ishikawa-yu@ncchd.go.jp (Y.I.); imadome-k@ncchd.go.jp (K.-I.I.)

**Keywords:** Epstein–Barr virus, infectious mononucleosis, EBV-associated lymphoma and epithelial cancer, EBV vaccine development, core fusion machinery, envelope glycoprotein, gH/gL, gB, neutralizing antibody

## Abstract

Epstein–Barr virus (EBV) is the primary cause of infectious mononucleosis and is strongly implicated in the etiology of multiple lymphoid and epithelial cancers. EBV core fusion machinery envelope proteins gH/gL and gB coordinately mediate EBV fusion and entry into its target cells, B lymphocytes and epithelial cells, suggesting these proteins could induce antibodies that prevent EBV infection. We previously reported that the immunization of rabbits with recombinant EBV gH/gL or trimeric gB each induced markedly higher serum EBV-neutralizing titers for B lymphocytes than that of the leading EBV vaccine candidate gp350. In this study, we demonstrated that immunization of rabbits with EBV core fusion machinery proteins induced high titer EBV neutralizing antibodies for both B lymphocytes and epithelial cells, and EBV gH/gL in combination with EBV trimeric gB elicited strong synergistic EBV neutralizing activities. Furthermore, the immune sera from rabbits immunized with EBV gH/gL or trimeric gB demonstrated strong passive immune protection of humanized mice from lethal dose EBV challenge, partially or completely prevented death respectively, and markedly decreased the EBV load in peripheral blood of humanized mice. These data strongly suggest the combination of EBV core fusion machinery envelope proteins gH/gL and trimeric gB is a promising EBV prophylactic vaccine.

## 1. Introduction

Epstein–Barr virus (EBV) is a gamma human herpesvirus that primarily infects B cells and epithelial cells. EBV is the primary cause of infectious mononucleosis [1,2]. There are ~125,000 new cases of infectious mononucleosis each year in the United States, and it is the most common cause of lost time for new army recruits [3,4,5]. Infectious mononucleosis could cause persistent fatigue for up to 6 months and cause severe neurologic, hematologic, or liver complications [2,3,6,7]. EBV is also the first human tumor virus discovered, and it has been strongly implicated in the etiology of multiple lymphoid and epithelial cancers, such as Burkitt lymphoma, Hodgkin lymphoma, non-Hodgkin lymphoma, post-transplant lymphoproliferative disorder, nasopharyngeal carcinoma, and gastric carcinoma [8,9,10,11]. Overall, EBV-associated cancers account for over 200,000 new cases of cancer and cause 150,000 deaths worldwide each year [3,8,10]. Patients undergoing solid organ or stem cell transplantation are at risk of developing uncontrolled B cell proliferation due to EBV reactivation, termed post-transplant lymphoproliferative disorder that can evolve into non-Hodgkin lymphoma, and a similar phenomenon also occurs in patients with AIDS [3,12,13,14]. A role for EBV has also been suggested in the pathogenesis of T and NK cell lymphomas, aggressive NK cell leukemia, and lymphoepithelioma-like carcinoma of the lung, salivary gland and thymus [15,16,17]. Many studies further suggest a possible role for EBV in the pathogenesis of several autoimmune diseases, including multiple sclerosis, systemic lupus erythematosus, rheumatoid arthritis and Sjogren’s syndrome [7,18].

Two distinct types of EBV are recognized: EBV-1 and EBV-2. The two types of EBV exhibit 70–85% sequence homology, and the differences between EBV-1 and EBV-2 isolates are largely confined to four latency proteins: EBNA-2, -3A, -3B, and -3C [19,20,21]. EBV is typically transmitted via saliva and contracted during infancy in developing countries, whereas in the developed world, it is typically contracted during adolescence [5,22]. The target cells of EBV are B lymphocytes and epithelial cells, and the mechanism by which EBV enters into host cells, is shared in many aspects by other members of the herpesvirus family [23,24,25,26,27]. Infection of B cells with EBV is initiated by binding of the EBV envelope protein gp350 to the complement receptor 2 (CR2)/CD21. Upon binding to B cell CR2, EBV gp42 interacts with the host cell surface MHC-II, leading to its association with the heterodimeric protein gH/gL. gH/gL then activates the EBV fusion protein gB, that directly mediates viral–host cell endosomal membrane fusion [24,25,26]. EBV infection of epithelial cells involves EBV BMRF2 binding to integrins, followed by gH/gL binding to integrins and ephrin receptor A2, triggering the activation of gB and fusion of the viral envelope to the plasma membrane of the epithelial cell [7,27,28,29,30,31,32,33,34]. Thus, EBV envelope glycoproteins gH/gL, gB and gp350 play key roles in the EBV infection of target cells, where gH/gL and gB constitute the “core fusion machinery” mediating fusion with the cell membrane [23,24,27]. The native conformation of EBV gB is a trimer, and EBV gH and gL naturally form a heterodimer [23,35]. EBV envelope proteins gH/gL and gB are essential for EBV infection of both B cells and epithelial cells, whereas gp350 is important for the efficient infection of B cells [23,24,27,32].

EBV vaccination holds great promise as an efficient way of prevention and management of EBV infection and associated diseases, and would have a considerable public health and economic impact [1,2,3,7]. Early research on EBV vaccine development was focused on gp350. Studies in non-human primates using gp350-based vaccination strategies have shown protection against EBV-induced lymphoma and EBV replication [36,37,38,39,40,41,42,43]. A phase II human clinical trial of a recombinant monomeric gp350 demonstrated 78% efficacy in preventing infectious mononucleosis, but failed to prevent EBV infection [44,45]. This was most likely due to gp350 only mediating the attachment of EBV to B cells, but with little role in the EBV infection of epithelial cells. Recently, additional EBV envelope proteins have been studied as EBV vaccine candidates; specifically, EBV gH/gL, gB and gp42 all were shown to induce EBV neutralizing antibodies [7,46]. Recombinant proteins have a highly favorable safety record as vaccines for human use and induce long-lasting memory responses. In combination with the new generation of lipid nanoparticle-based adjuvants, recombinant protein vaccines have demonstrated higher efficacy compared to live attenuated viral vaccine platforms [47,48]. We were the first to report that recombinant EBV gH/gL and gB proteins elicited markedly higher titers of EBV neutralization antibodies than gp350 after immunization in rabbits [46]. Compared to gp350, EBV gH/gL and trimeric gB elicited >20- and 18-fold higher EBV neutralizing antibodies respectively [46]. It was later demonstrated that EBV gH, gL, gB and gp42 in nanoparticle or virus-like particle forms also induced potent EBV neutralizing antibodies in mice and non-human primates [49,50].

Since the cotton-top tamarin, an endangered species, was banned for use in EBV research, humanized mice have been widely used for EBV studies and are currently considered the best model for recapitulating EBV-induced human B cell disease [51,52,53,54,55,56,57,58]. NOD/Shi-scid/IL-2Rγ^null^ (NOG) mice receive hematopoietic stem cell transplants from human cord blood reconstitute human B, T, and NK cells, macrophages, and dendritic cells. Inoculation of humanized NOG mice with ~1 × 10^3^ TD_50_ (50% transforming dose, high dose) of EBV causes B cell lymphoproliferation with histopathological findings and latent EBV gene expression similar to that observed in immunocompromised humans, and mortality by 10 weeks post-infection, whereas inoculation with a low dose of virus (~1 × 10^1^ TD_50_) resulted in apparently asymptomatic persistent infection [54,56]. The humanized NOG mouse is an excellent animal model for post-transplant lymphoproliferative disorder, a model of EBV associated lymphoid cancer caused by high dose EBV infection, which is also a powerful tool in the evaluation of EBV vaccine candidates in the prevention of EBV infection and associated cancers. 

In this study, we demonstrated that immunization of rabbits with EBV core fusion machinery proteins induced potent EBV neutralizing activities for protection of both B cells and epithelial cells, and showed that the EBV gH/gL and trimeric gB in combination elicited markedly high titers of EBV neutralizing activities than that induced by individual EBV proteins. Furthermore, the immune sera from rabbits immunized with EBV gH/gL or trimeric gB demonstrated strong passive immune protection of humanized mice from lethal dose EBV challenge, and markedly decreased the EBV load in peripheral blood of humanized mice.

## 2. Materials and Methods 

### 2.1. Cell Lines, EBV Strain, and Reagents

Raji and HeLa cell lines were purchased from American Type Culture Collection (ATCC, Manassas, VA, USA), and cultured using RPMI-1640 or EMEM medium respectively, supplemented with 10% fetal bovine serum. Plasmids p509 and p2670 expressing EBV BZLF1 and BALF4, and 293/2089 cells that were used to produce B95-8 virus expressing GFP were from Drs. Jefferey Cohen and Henri-Jacques Delecluse (National Institutes of Health, Maryland, USA and National Research Center for Environment and Health, Munich, Germany) [26,59,60]. Recombinant EBV gH/gL and trimeric gB were produced in our laboratory [46]. Both EBV gH/gL and trimeric gB proteins were expressed using Chinese hamster ovary cells (CHO), the recombinant EBV gH/gL protein comprising the full-length gL linked to gH extracellular domain as a fusion protein, and the recombinant EBV trimeric gB consisting of 3 homologous gB chains [46]. The polyclonal goat anti-rabbit IgG labeled with alkaline phosphatase was purchased from Southern Biotechnology (Birmingham, AL, USA), and alkaline phosphatase substrate, disodium p-nitrophenyl phosphate, was from Sigma-Aldrich (St. Louis, MO, USA).

### 2.2. Rabbit Immunizations

Groups of New Zealand white rabbits, 12–15 weeks old, five in each group, were injected subcutaneously with 25 µg of EBV recombinant protein gH/gL, trimeric gB or the combination of gH/gL and trimeric gB (25 µg each). The proteins were mixed with 13 µg of aluminum hydroxide (alum) and 50 µg CpG-ODN that function as adjuvants [46,61]. Rabbits were injected with adjuvants only as a negative control. Immunization was performed on day 1, and repeated on day 21 and day 42. Rabbit serum samples were taken before initial immunization, 10 days following each immunization and on day 52 [46]. These studies were conducted in accordance with the Guide for Care and Use of Laboratory Animals (Institute of Laboratory Animal Resources, NRC, WA, USA), and were approved by the USUHS Institutional Animal Care and Use Committee.

### 2.3. Measurement of Serum Titers of EBV gH/gL- and gB-Specific IgG

Antigen-specific antibodies against EBV gH/gL and gB were determined by ELISA as previously described [46,61,62,63]. Briefly, Immulon 4 ELISA plates were coated with 5 µg/mL of purified EBV recombinant protein gH/gL or trimeric gB in PBS overnight at 4 °C, followed by blocking with 1% bovine serum albumin (BSA) in PBS. Serum samples were serially diluted in three-fold with 1% BSA-PBS, and were added in the EBV gH/gL or trimeric gB protein coated ELISA plates and incubated overnight at 4 °C. The plates were then incubated with a polyclonal goat anti-rabbit IgG labeled with alkaline phosphatase for 1 h at 37 °C, followed by addition of 1 mg/mL alkaline phosphatase substrate (p-nitrophenyl phosphate, disodium) in Tris-HCl magnesium-sulfate buffer. The plates were washed with 0.1% Tween-20 in PBS between steps, and the absorbance at 450 nm was read on an ELISA reader.

### 2.4. Determination of Serum EBV Neutralizing Titers Using the B Lymphocyte Cell Line RAJI

Determination of serum in vitro EBV-neutralizing titers using Raji cells (EBV-positive human Burkitt lymphoma cell line) were performed as described [46,59]. Briefly, serial dilutions of serum samples were mixed for 2 h with GFP-EBV (B95-8/F) in 96-well plates, followed by the addition of Raji cells for 3 additional hours. The cells were then washed and re-cultured in medium alone for 3 days, fixed in paraformaldehyde and analyzed by flow cytometry for GFP+ Raji cells. The serum dilution that inhibits infectivity by 50% (IC_50_), based on reduction of the number of GFP+ cells, were calculated by non-linear regression analysis using Graph Pad Prizm 8. The EBV-neutralizing anti-gp350 mAb (72A1) was used as a positive control. Pre-immune sera and sera from rabbits injected with alum + CpG-ODN served as negative controls.

### 2.5. Determination of Serum EBV Neutralizing Titers Using the Epithelial Cell Line HeLa

Determination of serum in vitro EBV-neutralizing titers using HeLa cells (human cervical cancer epithelial cells) was similar to that of using Raji cells with modifications. Briefly, serial dilutions of serum samples were mixed with GFP-EBV in 96-well plates for 2 h, followed by adding the mixture to the HeLa cells for 3 additional hours. The cells were washed with media and cultured for 3 days. Following trypsinization and washing twice in PBS, the cells were fixed in paraformaldehyde and analyzed by flow cytometry for GFP+ HeLa cells. The serum dilution that inhibits infectivity by 50% (IC_50_), based on the reduction of the number of GFP+ cells, was calculated by non-linear regression analysis using Graph Pad Prizm 8. Pre-immune sera and sera from rabbits injected with alum + CpG-ODN served as negative controls.

### 2.6. Passive Immune Protection of Humanized Mice from Lethal Dose EBV Challenge

Humanized NOG mice that reconstitute with human B, T, NK cells, macrophages, and dendritic cells, are susceptible to EBV infection [54]. Inoculation with a low dose of virus (~1 × 10^1^ TD_50_) causes apparently asymptomatic persistent infection in humanized NOG mice, whereas inoculation with a high dose (~1 × 10^3^ TD_50_) of EBV causes B cell lymphoproliferation, with histopathological findings similar to that observed in immunocompromised humans, and results in mortality by 10 weeks post-infection [54].

Humanized NOG mice were made by intravenous injection of human CD34(+) HSCs isolated from cord blood (~1 × 10^4^–1.2 × 10^5^ cells/female mouse at 6–10-week-old). After the human hemato-immune system was reconstituted, 3 groups (*n* = 6) of humanized NOG mice were injected intraperitoneally with 300 μL of the day 52 pooled sera from rabbits immunized with EBV gH/gL, trimeric gB or adjuvant alone (alum + CpG-ODN) respectively. Two hours following intraperitoneal injection of rabbit sera, humanized NOG mice were infected intravenously with ~1 × 10^3^ TD_50_ of EBV (strain AKATA), a dose that induces B cell lymphoproliferation and fatality by 10 weeks. Peripheral blood was obtained every week following EBV infection, and EBV DNA in blood was quantified by real-time quantitative PCR [64].

### 2.7. Statistics

All the experiments were done at least three times for reproducibility. The serum titers of antigen specific IgG and the copy numbers of EBV DNA were expressed as geometric means +/− standard error of the mean. The serum titers of EBV neutralizing activity were expressed as geometric means +/− standard deviation of the mean. Statistical analyses were performed with GraphPad Prism 8, *p* values were determined by two-tailed Students *t*-test, and *p* < 0.05 was considered significant.

## 3. Results

### 3.1. Immunization of Rabbits with EBV gH/gL in Combination with Trimeric gB Led to Induction of High Serum Titers of gH/gL- and gB-Specific IgG with no Cross-Antigen Interference

**Figure 1:**
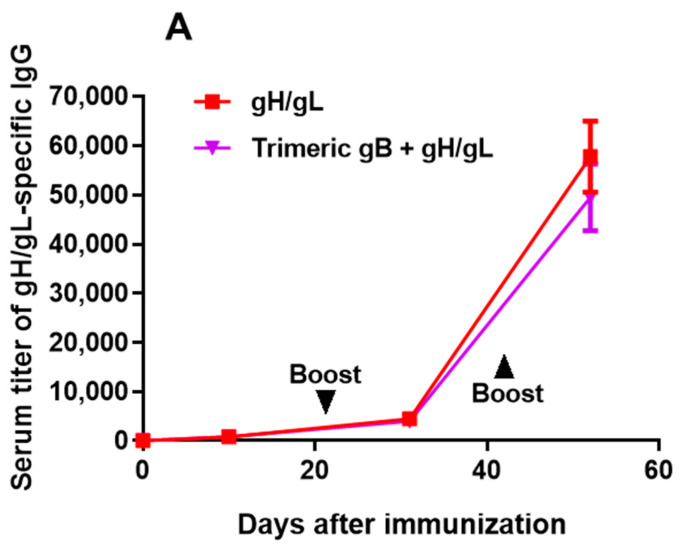

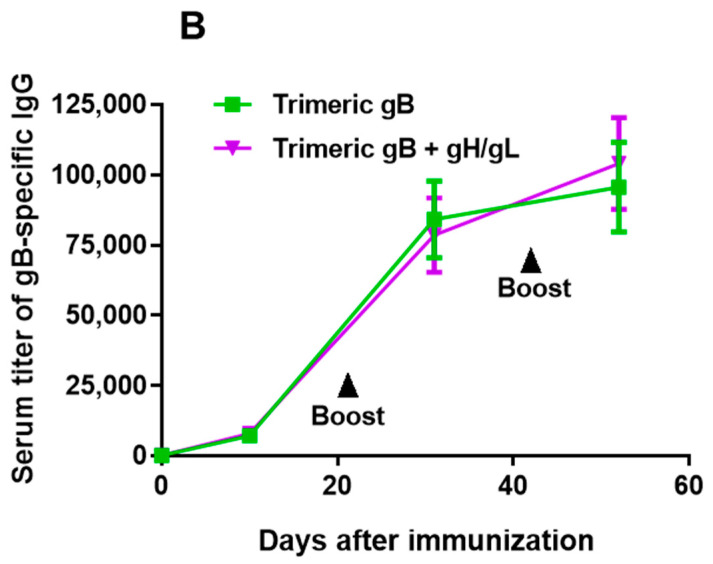
Immunization of rabbits with Epstein–Barr virus (EBV) gH/gL or trimeric gB recombinant protein induced high serum titers of antigen-specific IgG, without interference when EBV gH/gL and trimeric gB were used in combination. Groups of 12–15-week-old rabbits (*n* = 5), were subcutaneously immunized with 25 µg of recombinant EBV gH/gL, EBV trimeric gB or the combination of EBV gH/gL and trimeric gB (25 µg each) adjuvanted with alum + CpG-ODN, then boosted on days 21 and 42. Serum samples were obtained 10 days following each immunization, and on day 52 for measurement of serum titers of antigen-specific IgG by ELISA. (**A**) Serum titers of gH/gL-specific IgG. (**B**) Serum titers of gB-specific IgG.

Groups of 5 adult rabbits each were immunized subcutaneously with 25 µg of EBV recombinant protein gH/gL, trimeric gB or the combination of EBV gH/gL and trimeric gB, using alum + CpG-ODN as the adjuvant. Rabbits were then boosted in a similar fashion on days 21 and 42 post-immunization. As illustrated in Figure 1, similar to our previous report, EBV trimeric gB induced markedly augmented serum IgG response following the first booster immunization, whereas EBV gH/gL induced markedly increased serum IgG titers following the second booster immunization [46]. Rabbits immunized with EBV gH/gL or trimeric gB individually induced high serum IgG titers (~1:100,000) of antigen-specific antibodies after 3 immunizations. The serum titers of anti-gH/gL IgG induced by the immunization with the combination of EBV gH/gL and trimeric gB were not significantly different from the IgG titers induced by immunization with EBV gH/gL alone (Figure 1A). Similarly, the serum titers of anti-gB IgG induced by immunization with EBV trimeric gB in combination with gH/gL were not significantly different from the IgG titers induced by immunization with EBV trimeric gB alone (Figure 1B). These data indicate that the combined use of EBV gH/gL and trimeric gB for immunization does not result in cross-antigen interference for antibody production.

### 3.2. In Vitro Mixture of the EBV gH/gL Rabbit Immune Sera with EBV gB Rabbit Immune Sera Showed Synergistic EBV Neutralizing Activity for the B Lymphoma Cell Line Raji and the Epithelial Cell Line HeLa

**Figure 2:**
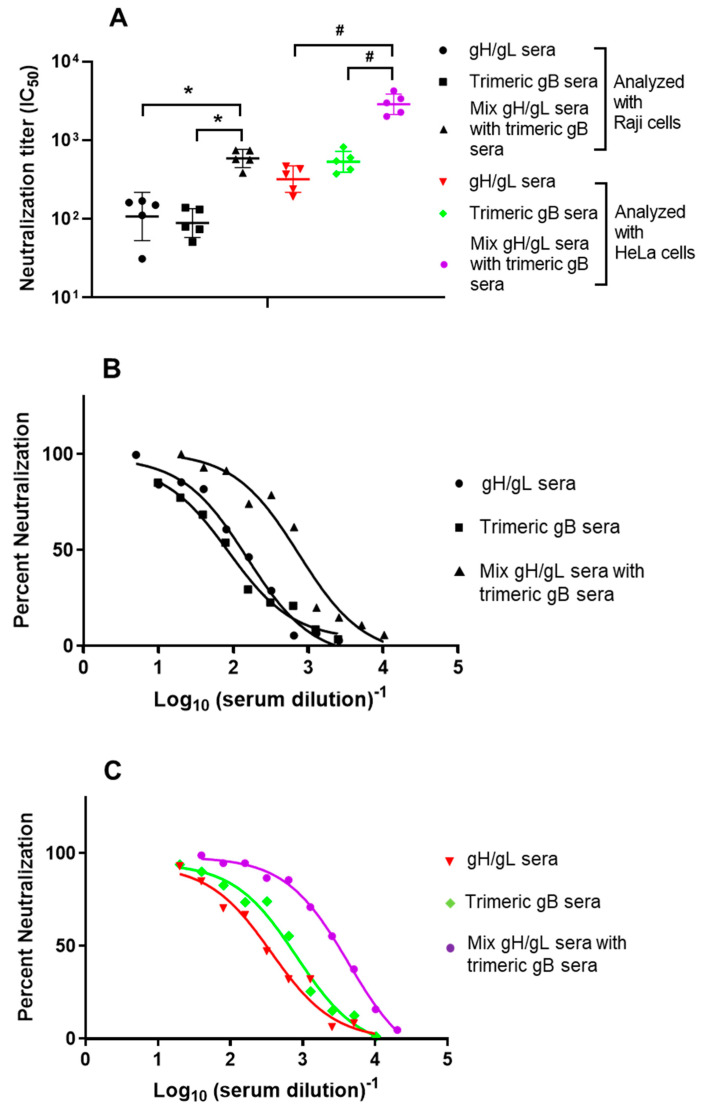
Mixing of the sera from rabbits immunized with EBV gH/gL and the sera from rabbits immunized with EBV trimeric gB in vitro showed synergistic neutralizing activity preventing EBV infection of the B lymphoma cell line Raji and the epithelial cell line HeLa. Day 52 immune sera from rabbits (*n* = 5) subcutaneously immunized three times with 25 µg of recombinant EBV gH/gL or EBV trimeric gB adjuvanted with alum + CpG-ODN were heat-inactivated. EBV gH/gL immune sera were mixed with EBV trimeric gB immune sera in equal volume. (**A**) Serum IC_50_ neutralizing titers were determined using Raji/HeLa cells and the EBV strain B95-8 GFP as indicated. Significance *, # *p* < 0.05 compared to the EBV neutralizing activities of the sera from rabbits immunized with individual envelope proteins. (**B**) Calculation of IC_50_ by non-linear regression analysis using Raji cells and EBV strain B95-8 GFP. (**C**) Calculation of IC_50_ by non-linear regression analysis using HeLa cells and EBV strain B95-8 GFP.

The EBV neutralizing activities of the sera from rabbits immunized with EBV gH/gL or EBV trimeric gB alone were directly compared to that of the gH/gL immune sera and trimeric gB immune sera mixed together in equal volume. Immune sera from rabbits immunized with EBV gH/gL or EBV trimeric gB alone showed high titers of EBV neutralizing activity analyzed using Raji cells (IC_50_ 88 and 107 respectively). The mixing of EBV gH/gL immune sera with EBV trimeric gB immune sera significantly increased EBV neutralization activity (IC_50_ 586, Figure 2A,B). The mixture of EBV gH/gL immune sera and EBV trimeric gB immune sera showed 5-fold and 6-fold increased EBV neutralizing activity respectively compared to the immune sera from rabbits immunized with EBV gH/gL or trimeric gB alone. Compared to the sum of the neutralization activities of EBV gH/gL immune sera and EBV trimeric gB immune sera, the mixture of EBV gH/gL immune sera and EBV trimeric gB immune sera showed 3~fold increased EBV neutralizing activity, clearly demonstrated synergistic effects in EBV neutralization activity (Figure 2A,B).

**Figure 3:**
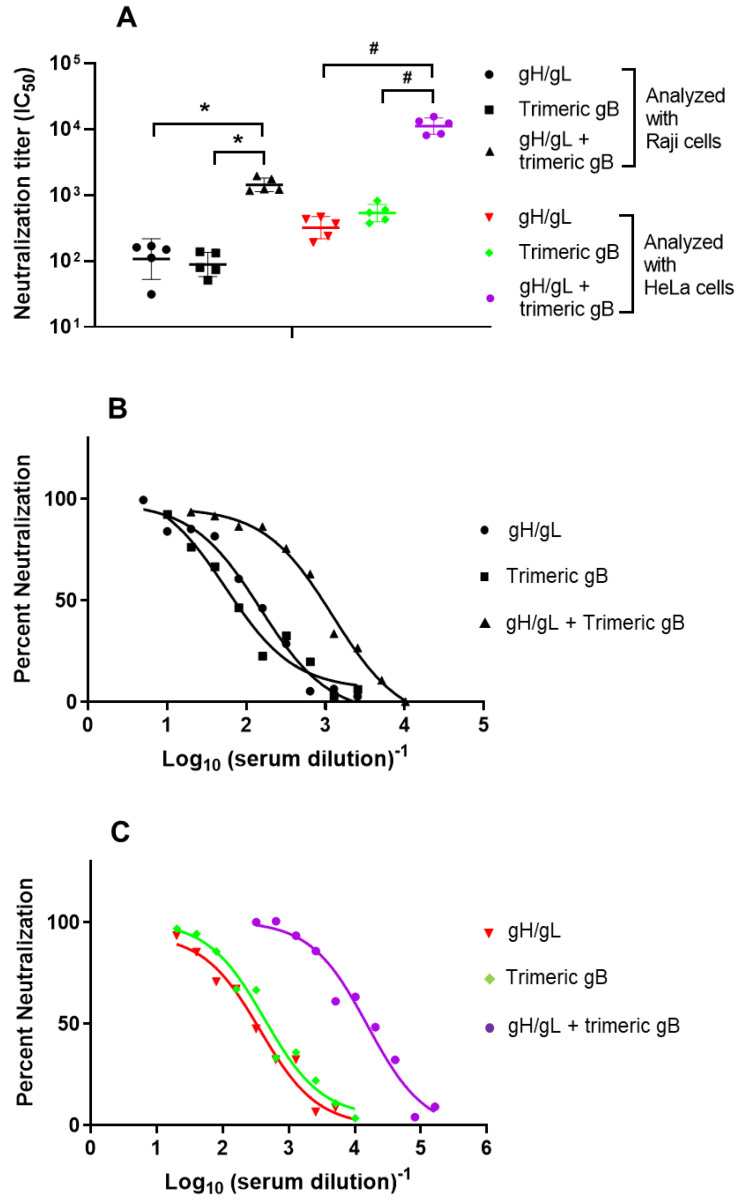
Immunization of rabbits with the combination of EBV gH/gL and trimeric gB demonstrated strong synergistic neutralizing activity preventing EBV infection of Raji cells and HeLa cells. Day 52 immune sera from rabbits (*n* = 5) subcutaneously immunized three times with 25 µg of recombinant EBV gH/gL, EBV trimeric gB or the combination of EBV gH/gL and trimeric gB (25 µg each) adjuvanted with alum + CpG-ODN were heat-inactivated. (**A**) Serum IC_50_ neutralizing titers were determined using Raji/HeLa cells and EBV strain B95-8 GFP as indicated. Significance *, # *p* < 0.05 compared to the EBV neutralizing activities of the sera from rabbits immunized with individual envelope proteins. (**B**) Calculation of IC_50_ by non-linear regression analysis using Raji cells and EBV strain B95-8 GFP. (**C**) Calculation of IC_50_ by non-linear regression analysis using HeLa cells and EBV strain B95-8 GFP.

The human SVKCR2 cell line has been used to determine EBV neutralizing activities protecting epithelial cells [50]. Since SVKCR2 cells express human complement receptor 2 (CR2), there has been concern that the EBV neutralizing titers for protection of epithelial cells determined using SVKCR2 cells may not show the actual neutralizing activities, as epithelial cells do not express CR2. EBV strains expressing high level gp110 could infect epithelial cells such as HeLa cells and gastric epithelial cells, and these cell lines might be better target cells for analyzing the EBV neutralizing activities protecting epithelial cells [26,60]. In this study, we used HeLa cells to determine the EBV neutralizing activities protecting epithelial cells. The EBV neutralizing activities analyzed using HeLa cells were higher than that of Raji cells, EBV gH/gL and EBV trimeric gB induced EBV neutralizing activities of IC_50_ 321 and 533 respectively (Figure 2A,C). The mixture of EBV gH/gL immune sera with EBV trimeric gB immune sera showed significantly increased EBV neutralization activity (IC_50_ 2878), which was 9-fold and 5-fold higher respectively compared to the immune sera from rabbits immunized with EBV gH/gL or EBV trimeric gB alone (Figure 2A,C). Compared to the sum of the neutralization activities of EBV gH/gL immune sera and EBV trimeric gB immune sera, the mixture of EBV gH/gL and EBV trimeric gB immune sera showed a 3.5-fold increase of EBV neutralizing activity analyzed using HeLa cells, demonstrating synergistic effects in EBV neutralization activity for epithelial cells (Figure 3A,C).

### 3.3. Immunization with the Combination of EBV gH/gL and Trimeric gB Elicited Strong Synergistic EBV Neutralizing Activity for the B Lymphoma Cell Line Raji and the Epithelial Cell Line HeLa

As the mixture of EBV gH/gL rabbit immune sera with EBV trimeric gB immune sera demonstrated synergistic neutralizing activity against EBV, we determined the EBV neutralizing activity induced by immunization with the combination of EBV gH/gL and trimeric gB, components of the EBV core fusion machinery. As shown in Figure 3A,B, immunization with the combination of EBV gH/gL and EBV trimeric gB elicited markedly higher EBV neutralizing activity analyzed using Raji cells (IC_50_ 1432), which was ~13-fold and ~16-fold higher than that induced by EBV gH/gL (IC_50_ 107) or EBV trimeric gB (IC_50_ 88) alone respectively.

EBV neutralizing activity of the sera from rabbits immunized with EBV gH/gL, EBV trimeric gB or the combination of EBV gH/gL and EBV trimeric gB were also analyzed using HeLa cells. Similar to analysis with Raji cells, immunization with the combination of EBV gH/gL and EBV trimeric gB markedly increased the EBV neutralizing activity for epithelial cells (IC_50_ 11194) compared to that of the immunization with EBV gH/gL or EBV trimeric gB alone, analyzed using HeLa cells (Figure 3A,C). Relative to the sera from rabbits immunized with EBV gH/gL alone (IC_50_ 321), the immune sera from rabbits immunized with the combination of EBV gH/gL and EBV trimeric gB showed ~35-fold increased EBV neutralizing activity for epithelial cells (Figure 3A,C). Similarly, the immune sera from rabbits immunized with the combination of EBV gH/gL and EBV trimeric gB demonstrated ~21-fold increased EBV neutralizing activity for epithelial cells compared to that of the sera from rabbits immunized with EBV trimeric gB alone (IC_50_ 533) (Figure 3A,C).

### 3.4. Immune Sera from Rabbits Immunized with EBV gH/gL or EBV Trimeric gB Protected Humanized Mice from Death Caused by High Dose EBV Challenge

Humanized NOG mice reconstitute with human B, T cells, macrophages, and dendritic cells, and can recapitulate key aspects of EBV infection in humans [54]. Intravenous injection of humanized NOG mice with ~1 × 10^3^ TD_50_ (50% transforming dose) of EBV causes B cell lymphoproliferation with histopathological findings and latent EBV gene expression similar to that observed in immunocompromised humans, and result in mortality by 10 weeks post-infection [54]. To determine whether the antibodies induced by EBV core fusion machinery proteins gH/gL or gB could confer passive protection against EBV, humanized NOG mice were injected intraperitoneally with 300 µL of pooled sera from rabbits immunized with EBV gH/gL or EBV trimeric gB, followed by intravenous infection with 1 × 10^3^ TD_50_ EBV (high dose). Humanized NOG mice injected intraperitoneally with 300 µL of pooled sera from rabbits injected with adjuvant alum + CpG-ODN only served as a negative control. 

**Figure 4:**
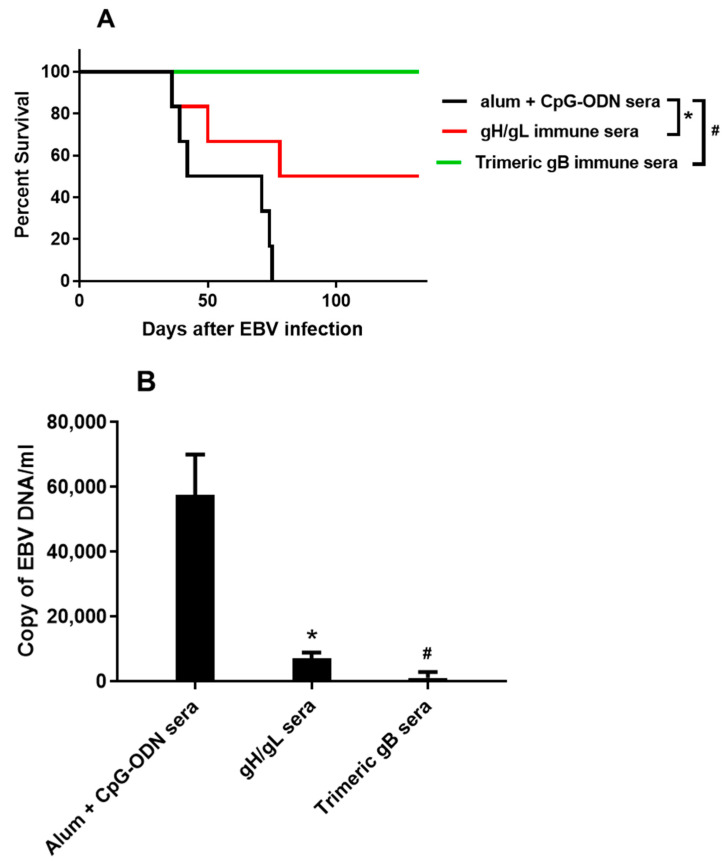
Immune sera from rabbits immunized with EBV gH/gL or EBV trimeric gB protected humanized NOG mice from death caused by high dose EBV infection, and markedly decreased the EBV load in peripheral blood. Humanized NOG mice were made by intravenous injection of human CD34+ HSCs isolated from cord blood. After the human hemato-immune system was reconstituted, 3 groups (*n* = 6) of humanized NOG mice were injected intraperitoneally with 300 µL of the day 52 pooled sera from rabbits immunized with EBV gH/gL, EBV trimeric gB or adjuvant alone (alum + CpG-ODN) respectively. Two hours following intraperitoneal injection of rabbit sera, humanized mice were infected intravenously with ~1 × 10^3^ TD_50_ of EBV (AKATA), a dose that induces B cell lymphoproliferation and fatality by 10 weeks (67). (**A**) Survival curve 132 days post-EBV infection. (**B**) Copy number of EBV DNA in peripheral blood of humanized mice 35 days after high dose EBV infection. Significance *, # *p* < 0.05 compared to the humanized mice receiving the sera from rabbits injected with adjuvant alone.

Seventy-five days after the intravenous infection with high dose EBV, all the humanized NOG mice receiving the pooled sera from rabbits injected with adjuvant only were dead. In contrast, half (3/6) of the humanized NOG mice receiving pooled EBV gH/gL rabbit immune sera survived, and all (6/6) of the humanized NOG mice receiving pooled EBV trimeric gB rabbit immune sera survived 132 days after high dose EBV infection (Figure 4A). Quantitative PCR analysis of the EBV DNA in peripheral blood 35 days after high dose EBV infection showed high copy number of EBV DNA in the humanized NOG mice receiving the pooled sera from rabbits injected with adjuvant only (~57,000/mL blood), whereas in the humanized NOG mice receiving pooled EBV gH/gL rabbit immune sera the EBV DNA in peripheral blood decreased 87%, and in the humanized NOG mice receiving pooled EBV trimeric gB rabbit immune sera the EBV DNA in peripheral blood decreased 98% (Figure 4B).

## 4. Discussion

EBV is the first human tumor virus identified. EBV not only causes infectious mononucleosis, it is also strongly associated with epithelial cell cancers such as nasopharyngeal cancer (NPC), gastric cancer as well as lymphoid cancers such as Burkitt lymphoma, Hodgkin lymphoma and post transplantation lymphoproliferative disorder (PTLD) [8,9,10,11,12,13,14]. NPC is endemic in southeast Asia, and the vast majority are the nonkeratinized type, accounting for 80,000 new cases each year worldwide [10,65,66]. Nonkeratinized NPC display a lymphoepithelial-like (LEL) appearance with a marked lymphocytic infiltration, which is 100% EBV positive [66]. About 10% of gastric cancers are associated with EBV infection, have a similar LEL pathological change and are 100% EBV positive, accounting for about 83,000 new cases each year [66,67,68]. Essentially, all Burkitt lymphoma in equatorial Africa and in Papua New Guinea, are EBV genome-positive, accounting for 7000 new cases each year [69,70,71]. Hodgkin lymphoma is also strongly associated with EBV, especially the mixed cellularity subtype, of which 80–90% are EBV positive [72,73,74,75]. PTLD is another example that EBV plays a critical role in cancer pathogenesis, all the cases of PTLD are EBV positive, and adoptive transfer of EBV specific T cells could prevent or cure the disease [76,77,78,79,80]. The role of EBV in cancer pathogenesis has also been confirmed in animal models, as the inoculation of cotton top tamarins or humanized mice with high titers of EBV results in the development of B-cell lymphomas and lymphoproliferative disease that are seen in humans [36,37,38,39,40,41,42,43,54,58].

An EBV prophylactic vaccine holds great promise for prevention of cancers caused by EBV infection, as has been the case for prophylactic vaccines against human papilloma virus and hepatitis B virus that cause ~600,000 and ~400,000 cases of new cancers each year respectively [10,81]. Early efforts in EBV vaccine development were focused on gp350. EBV gp350 is the most abundant EBV envelope protein, and about half of the EBV neutralizing activity in EBV seropositive human sera is against gp350 [7,50]. Immunization with purified or recombinant gp350 was shown to protect cotton top tamarins from lymphoma caused by EBV infection, and similar results were reported with adenovirus or vaccinia virus expressing gp350 [36,37,38,39,40,41,42,43]. Phase I and II clinical studies of a recombinant gp350 produced in Chinese hamster ovary cells showed that the recombinant gp350 induced neutralizing antibodies in humans in 70% of the subjects, and reduced the rate of infectious mononucleosis by 78% in the vaccinated subjects but did not prevent EBV infection [44,45]. This is most likely because gp350 is not strictly essential for EBV virus infection of B cells but only important for efficient infection, as well as the inability of gp350 induced antibodies to protect against EBV infection of epithelial cells [23,24,27,32,82].

The target cells of EBV are mainly B cells and epithelial cells, and EBV requires multiple envelope proteins for cell entry. EBV infection of B cells requires envelope proteins gp350, gH, gL, gB and gp42, whereas EBV infection of epithelial cells requires envelope proteins BMFR2, gH, gL and gB [7,24,25,26,32]. Our group was the first to report in 2016 that recombinant EBV gH/gL and gB proteins induce markedly higher EBV neutralizing antibodies compared to gp350 [46]. This was confirmed by the recently published study by Bu et al. that EBV gH/gL or gH/gL/gp42 nanoparticles induced potent neutralizing antibody responses in mice and non-human primates, which blocked EBV-target cell fusion and prevented EBV infection of B cells and epithelial cells [50]. Though these nanoparticle EBV vaccine candidates induced 10- to 1000-fold higher titers of neutralizing antibodies compared to that of soluble proteins, as the gH, gL and/or gp42 proteins were highly packed into the nanoparticles, the expression of native conformational epitopes of these EBV envelope proteins could be prevented [50]. It was reported that virus like particles and nanoparticles could induce quantitatively high antibody responses whereas recombinant proteins expressing native epitopes could elicit antibody responses that are high both quantitatively and qualitatively [83,84,85,86]. This has been confirmed with the herpes virus recombinant envelope protein vaccine candidates produced in our laboratory including EBV gH/gL and EBV trimeric gB [46,61,87]. EBV gp350 and BMRF2 are required for EBV attachment to B cells and epithelial cells respectively, whereas EBV gH/gL and gB constitute the core fusion machinery, which play the critical roles for EBV fusion and entry into all target cells, thus making EBV gH/gL and gB more promising as prophylactic EBV vaccine candidates [23,24,27]. As EBV gH/gL and gB could both elicit neutralizing antibodies, the presence of gH/gL neutralizing antibodies and gB neutralizing antibodies simultaneously would most likely exhibit synergistic effects [7,23,32]. As in this study, mixing EBV gH/gL anti-sera with gB anti-sera showed synergistic neutralizing activity as gH/gL and gB antibodies block different steps in EBV infection of target cells including both B cells and epithelial cells.

In this study, immunization with the combination of EBV gH/gL and trimeric gB induced high titers of antigen-specific antibodies against EBV gH/gL and gB that were no different compared to that of immunization with EBV gH/gL or EBV trimeric gB alone. This is consistent with our previous study as well as studies by other investigators that immunization with combinations of herpes virus envelope proteins induced high titers of antigen-specific antibodies without interference in the induction of individual protein-specific antibodies [61,88,89]. Immunization with the combination of EBV gH/gL and EBV trimeric gB elicited markedly higher EBV neutralizing activities for both B cells and epithelial cells as compared to that induced by immunization with EBV gH/gL or EBV trimeric gB alone, demonstrating strong synergistic effects of EBV core fusion envelope proteins in elicitation of neutralizing antibodies. The strong synergistic effects are most likely due to the sequential coordination of these envelope proteins in mediating EBV entry and infection of target cells. These data suggest that the combination of EBV core fusion machinery envelope proteins gH/gL and trimeric gB could be an ideal EBV prophylactic vaccine.

To determine whether the EBV neutralizing antibodies induced by EBV core fusion machinery envelope proteins gH/gL and trimeric gB are protective against EBV infection in vivo, we performed passive immune protection studies using humanized mice. Consistent with previous studies, all the humanized mice injected with the pooled sera from rabbits injected with adjuvants alone died 75 days after high dose EBV challenge [54]. In contrast, the pooled sera from rabbits immunized with EBV trimeric gB completely protected humanized mice from death caused by lethal dose EBV infection, whereas the pooled sera from rabbits immunized with EBV gH/gL showed partial protection. Compared to the pooled sera from rabbits injected with adjuvants alone, the pooled immune sera from rabbits immunized with EBV gH/gL or EBV trimeric gB markedly decreased EBV load in peripheral blood of humanized mice 35 days after EBV challenge. EBV gB directly mediates virus fusion with the host cell membrane, which is the last and most important step of EBV entry into host cells, whereas EBV gH/gL mediates virus binding to the host cells [7,23,32]. This may explain why EBV trimeric gB immune sera demonstrated significantly better protection of the humanized mice than the EBV gH/gL immune sera. A similar result was also obtained with another human herpesvirus, human cytomegalovirus (HCMV), where HCMV gB proved to be the most effective among several vaccine candidates in eliciting immune response for host protection [85,86,90]. As immunization with the combination of EBV gH/gL and EBV trimeric gB induced strong synergistic EBV neutralizing activities, the sera from rabbits immunized with the combination of EBV gH/gL and EBV trimeric gB would demonstrate significantly better passive immune protection than that of the sera from rabbits immunized with EBV trimeric gB alone. This will be conducted in our future studies, together with an active vaccination study with the combination of EBV gH/gL and EBV trimeric gB using humanized DRAGA mice (HLA-A2.HLA-DR4.Rag1KO.IL2RγcKO.NOD), which most likely would provide sterilizing immunization [91,92,93].

## 5. Conclusions

EBV is the first human tumor virus discovered, and it has been strongly implicated in the etiology of multiple lymphoid and epithelial cancers, such as Burkitt lymphoma, Hodgkin lymphoma, non-Hodgkin lymphoma, post-transplant lymphoproliferative disorder, nasopharyngeal carcinoma, and gastric carcinoma. EBV associated cancers account for over 200,000 new cases of cancer and cause 150,000 deaths world-wide each year. EBV is also the primary cause of infectious mononucleosis, and is associated with the pathogenesis of autoimmune diseases, including multiple sclerosis, systemic lupus erythematosus and rheumatoid arthritis. Thus, EBV vaccination holds great promise as an efficient way of prevention and management of EBV infection and associated diseases, and would have a considerable public health and economic impact.

The target cells of EBV are B lymphocytes and epithelial cells, and EBV requires multiple envelope proteins for cell entry where gH/gL and gB constitute the “core fusion machinery” mediating fusion with the cell membrane. EBV gH/gL and gB proteins have been shown to induce markedly higher EBV neutralizing antibodies compared to the leading EBV vaccine candidate gp350, block EBV-target cell fusion, and prevent EBV infection of both B lymphocytes and epithelial cells. In this study, we further demonstrated that the immune sera from rabbits immunized with EBV gH/gL or trimeric gB conferred strong passive immune protection of humanized mice from lethal dose EBV challenge, partially or completely prevented death respectively, and markedly decreased the EBV load in peripheral blood of humanized mice. 

Similar to other members of the herpesvirus family such as human cytomegalovirus and human simplex virus, EBV gH/gL and gB are essential for the virus infection of its target cells. As EBV gH/gL and gB could both elicit neutralizing antibodies, the presence of gH/gL neutralizing antibodies and gB neutralizing antibodies simultaneously would block the sequential coordination of these envelope proteins in mediating EBV entry and infection of target cells. As shown in this study, immunization with the combination of EBV gH/gL and trimeric gB elicited markedly higher EBV neutralizing activities for both B cells and epithelial cells as compared to that induced by immunization with EBV gH/gL or trimeric gB alone, demonstrating strong synergistic effects of EBV core fusion envelope proteins in elicitation of neutralizing antibodies. Recombinant proteins have a highly favorable safety record as vaccines and induce long-lasting memory responses. In combination with the new generation of lipid nanoparticle based adjuvants, recombinant protein vaccines have demonstrated higher efficacy compared to live attenuated viral vaccine platforms. These data suggest that the combination of recombinant EBV core fusion machinery envelope proteins gH/gL and trimeric gB could be an ideal EBV prophylactic vaccine, where native epitopes could elicit high titer antibody responses both quantitatively and qualitatively.

## Data Availability

The data presented in this study are available on request from the corresponding author.

## References

[1] Cohen J.I., Fauci A.S., Varmus H., Nabel G.J. (2011). Epstein-Barr virus: An important vaccine target for cancer prevention. Sci. Transl. Med..

[2] Vetsika E.K., Callan M. (2004). Infectious mononucleosis and Epstein-Barr virus. Expert Rev. Mol. Med..

[3] Cohen J.I., Mocarski E.S., Raab-Traub N., Corey L., Nabel G.J. (2013). The need and challenges for development of an Epstein-Barr virus vaccine. Vaccine.

[4] Luzuriaga K., Sullivan J.L. (2010). Infectious mononucleosis. N. Engl. J. Med..

[5] Hallee T.J., Evans A.S., Niederman J.C., Brooks C.M., Voegtly J.H. (1974). Infectious mononucleosis at the United States Military Academy. A prospective study of a single class over four years. Yale J. Biol. Med..

[6] Rea T.D., Russo J.E., Katon W., Ashley R.L., Buchwald D.S. (2001). Prospective study of the natural history of infectious mononucleosis caused by Epstein-Barr virus. J. Am. Board Fam. Pract..

[7] Cohen J.I. (2018). Vaccine Development for Epstein-Barr Virus. Adv. Exp. Med. Biol..

[8] Neparidze N., Lacy J. (2014). Malignancies associated with epstein-barr virus: Pathobiology, clinical features, and evolving treatments. Clin. Adv. Hematol. Oncol..

[9] Fukayama M. (2010). Epstein-Barr virus and gastric carcinoma. Pathol. Int..

[10] Parkin D.M. (2006). The global health burden of infection-associated cancers in the year 2002. Int. J. Cancer.

[11] Hjalgrim H., Askling J., Sorensen P., Madsen M., Rosdahl N., Storm H.H., Hamilton-Dutoit S., Eriksen L.S., Frisch M., Ekbom A. (2000). Risk of Hodgkin’s disease and other cancers after infectious mononucleosis. J. Natl. Cancer Inst..

[12] Dharnidharka V.R. (2018). Comprehensive review of post-organ transplant hematologic cancers. Am. J. Transplant..

[13] Al-Mansour Z., Nelson B.P., Evens A.M. (2013). Post-transplant lymphoproliferative disease (PTLD): Risk factors, diagnosis, and current treatment strategies. Curr. Hematol. Malig. Rep..

[14] AlDabbagh M.A., Gitman M.R., Kumar D., Humar A., Rotstein C., Husain S. (2017). The Role of Antiviral Prophylaxis for the Prevention of Epstein-Barr Virus-Associated Posttransplant Lymphoproliferative Disease in Solid Organ Transplant Recipients: A Systematic Review. Am. J. Transplant..

[15] Kimura H., Ito Y., Kawabe S., Gotoh K., Takahashi Y., Kojima S., Naoe T., Esaki S., Kikuta A., Sawada A. (2012). EBV-associated T/NK-cell lymphoproliferative diseases in nonimmunocompromised hosts: Prospective analysis of 108 cases. Blood.

[16] Song S.Y., Kim W.S., Ko Y.H., Kim K., Lee M.H., Park K. (2002). Aggressive natural killer cell leukemia: Clinical features and treatment outcome. Haematologica.

[17] Iezzoni J.C., Gaffey M.J., Weiss L.M. (1995). The role of Epstein-Barr virus in lymphoepithelioma-like carcinomas. Am. J. Clin. Pathol..

[18] Villegas E., Santiago O., Sorlozano A., Gutierrez J. (2010). New strategies and patent therapeutics in EBV-associated diseases. Mini Rev. Med. Chem..

[19] Dambaugh T., Wang F., Hennessy K., Woodland E., Rickinson A., Kieff E. (1986). Expression of the Epstein-Barr virus nuclear protein 2 in rodent cells. J. Virol..

[20] Sample J., Young L., Martin B., Chatman T., Kieff E., Rickinson A., Kieff E. (1990). Epstein-Barr virus types 1 and 2 differ in their EBNA-3A, EBNA-3B, and EBNA-3C genes. J. Virol..

[21] Rowe M., Young L.S., Cadwallader K., Petti L., Kieff E., Rickinson A.B. (1989). Distinction between Epstein-Barr virus type A (EBNA 2A) and type B (EBNA 2B) isolates extends to the EBNA 3 family of nuclear proteins. J. Virol..

[22] Niederman J.C., McCollum R.W., Henle G., Henle W. (1968). Infectious mononucleosis. Clinical manifestations in relation to EB virus antibodies. JAMA.

[23] Connolly S.A., Jackson J.O., Jardetzky T.S., Longnecker R. (2011). Fusing structure and function: A structural view of the herpesvirus entry machinery. Nat. Rev. Microbiol..

[24] Hutt-Fletcher L.M. (2007). Epstein-Barr virus entry. J. Virol..

[25] Shannon-Lowe C., Rowe M. (2014). Epstein Barr virus entry; kissing and conjugation. Curr. Opin. Virol..

[26] Neuhierl B., Feederle R., Hammerschmidt W., Delecluse H.J. (2002). Glycoprotein gp110 of Epstein-Barr virus determines viral tropism and efficiency of infection. Proc. Natl. Acad. Sci. USA.

[27] Heldwein E.E., Krummenacher C. (2008). Entry of herpesviruses into mammalian cells. Cell Mol. Life Sci..

[28] Jiang R., Gu X., Nathan C.O., Hutt-Fletcher L. (2008). Laser-capture microdissection of oropharyngeal epithelium indicates restriction of Epstein-Barr virus receptor/CD21 mRNA to tonsil epithelial cells. J. Oral Pathol. Med..

[29] Birkenbach M., Tong X., Bradbury L.E., Tedder T.F., Kieff E. (1992). Characterization of an Epstein-Barr virus receptor on human epithelial cells. J. Exp. Med..

[30] Maruo S., Yang L., Takada K. (2001). Roles of Epstein-Barr virus glycoproteins gp350 and gp25 in the infection of human epithelial cells. J. Gen. Virol..

[31] Fingeroth J.D., Diamond M.E., Sage D.R., Hayman J., Yates J.L. (1999). CD21-Dependent infection of an epithelial cell line, 293, by Epstein-Barr virus. J. Virol..

[32] Hutt-Fletcher L.M. (2014). Epstein-Barr virus replicating in epithelial cells. Proc. Natl. Acad. Sci. USA.

[33] Tsao S.W., Tsang C.M., Pang P.S., Zhang G., Chen H., Lo K.W. (2012). The biology of EBV infection in human epithelial cells. Semin. Cancer Biol..

[34] Li Q.X., Young L.S., Niedobitek G., Dawson C.W., Birkenbach M., Wang F., Rickinson A.B. (1992). Epstein-Barr virus infection and replication in a human epithelial cell system. Nature.

[35] Backovic M., Longnecker R., Jardetzky T.S. (2009). Structure of a trimeric variant of the Epstein-Barr virus glycoprotein B. Proc. Natl. Acad. Sci. USA.

[36] Sashihara J., Hoshino Y., Bowman J.J., Krogmann T., Burbelo P.D., Coffield V.M., Kamrud K., Cohen J.I. (2011). Soluble rhesus lymphocryptovirus gp350 protects against infection and reduces viral loads in animals that become infected with virus after challenge. PLoS Pathog..

[37] Morgan A.J., Epstein M.A., North J.R. (1984). Comparative immunogenicity studies on Epstein-Barr virus membrane antigen (MA) gp340 with novel adjuvants in mice, rabbits, and cotton-top tamarins. J. Med. Virol..

[38] Morgan A.J., Allison A.C., Finerty S., Scullion F.T., Byars N.E., Epstein M.A. (1989). Validation of a first-generation Epstein-Barr virus vaccine preparation suitable for human use. J. Med. Virol..

[39] Finerty S., Tarlton J., Mackett M., Conway M., Arrand J.R., Watkins P.E., Morgan A.J. (1992). Protective immunization against Epstein-Barr virus-induced disease in cottontop tamarins using the virus envelope glycoprotein gp340 produced from a bovine papillomavirus expression vector. J. Gen. Virol..

[40] Cox C., Naylor B.A., Mackett M., Arrand J.R., Griffin B.E., Wedderburn N. (1998). Immunization of common marmosets with Epstein-Barr virus (EBV) envelope glycoprotein gp340: Effect on viral shedding following EBV challenge. J. Med. Virol..

[41] Mackett M., Cox C., Pepper S.D., Lees J.F., Naylor B.A., Wedderburn N., Arrand J.R. (1996). Immunisation of common marmosets with vaccinia virus expressing Epstein-Barr virus (EBV) gp340 and challenge with EBV. J. Med. Virol..

[42] Ragot T., Finerty S., Watkins P.E., Perricaudet M., Morgan A.J. (1993). Replication-defective recombinant adenovirus expressing the Epstein-Barr virus (EBV) envelope glycoprotein gp340/220 induces protective immunity against EBV-induced lymphomas in the cottontop tamarin. J. Gen. Virol..

[43] Morgan A.J., Mackett M., Finerty S., Arrand J.R., Scullion F.T., Epstein M.A. (1988). Recombinant vaccinia virus expressing Epstein-Barr virus glycoprotein gp340 protects cottontop tamarins against EB virus-induced malignant lymphomas. J. Med. Virol..

[44] Sokal E.M., Hoppenbrouwers K., Vandermeulen C., Moutschen M., Leonard P., Moreels A., Haumont M., Bollen A., Smets F., Denis M. (2007). Recombinant gp350 vaccine for infectious mononucleosis: A phase 2, randomized, double-blind, placebo-controlled trial to evaluate the safety, immunogenicity, and efficacy of an Epstein-Barr virus vaccine in healthy young adults. J. Infect. Dis..

[45] Moutschen M., Leonard P., Sokal E.M., Smets F., Haumont M., Mazzu P., Bollen A., Denamur F., Peeters P., Dubin G. (2007). Phase I/II studies to evaluate safety and immunogenicity of a recombinant gp350 Epstein-Barr virus vaccine in healthy adults. Vaccine.

[46] Cui X., Cao Z., Chen Q., Arjunaraja S., Snow A.L., Snapper C.M. (2016). Rabbits immunized with Epstein-Barr virus gH/gL or gB recombinant proteins elicit higher serum virus neutralizing activity than gp350. Vaccine.

[47] Lal H., Cunningham A.L., Heineman T.C. (2015). Adjuvanted Herpes Zoster Subunit Vaccine in Older Adults. N. Engl. J. Med..

[48] Cunningham A.L., Heineman T.C., Lal H., Godeaux O., Chlibek R., Hwang S.J., McElhaney J.E., Vesikari T., Andrews C., Choi W.S. (2018). Immune Responses to a Recombinant Glycoprotein E Herpes Zoster Vaccine in Adults Aged 50 Years or Older. J. Infect. Dis..

[49] Perez E.M., Foley J., Tison T., Silva R., Ogembo J.G. (2017). Novel Epstein-Barr virus-like particles incorporating gH/gL-EBNA1 or gB-LMP2 induce high neutralizing antibody titers and EBV-specific T-cell responses in immunized mice. Oncotarget.

[50] Bu W., Joyce M.G., Nguyen H., Banh D.V., Aguilar F., Tariq Z., Yap M.L., Tsujimura Y., Gillespie R.A., Tsybovsky Y. (2019). Immunization with Components of the Viral Fusion Apparatus Elicits Antibodies That Neutralize Epstein-Barr Virus in B Cells and Epithelial Cells. Immunity.

[51] Berges B.K., Tanner A. (2014). Modelling of human herpesvirus infections in humanized mice. J. Gen. Virol..

[52] Cocco M., Bellan C., Tussiwand R., Corti D., Traggiai E., Lazzi S., Mannucci S., Bronz L., Palummo N., Ginanneschi C. (2008). CD34+ cord blood cell-transplanted Rag2-/- gamma(c)-/- mice as a model for Epstein-Barr virus infection. Am. J. Pathol..

[53] Kuwana Y., Takei M., Yajima M., Imadome K., Inomata H., Shiozaki M., Ikumi N., Nozaki T., Shiraiwa H., Kitamura N. (2011). Epstein-Barr virus induces erosive arthritis in humanized mice. PLoS ONE.

[54] Yajima M., Imadome K., Nakagawa A., Watanabe S., Terashima K., Nakamura H., Ito M., Shimizu N., Honda M., Yamamoto N. (2008). A new humanized mouse model of Epstein-Barr virus infection that reproduces persistent infection, lymphoproliferative disorder, and cell-mediated and humoral immune responses. J. Infect. Dis..

[55] Traggiai E., Chicha L., Mazzucchelli L., Bronz L., Piffaretti J.C., Lanzavecchia A., Manz M.G. (2004). Development of a human adaptive immune system in cord blood cell-transplanted mice. Science.

[56] Seung E., Tager A.M. (2013). Humoral immunity in humanized mice: A work in progress. J. Infect. Dis..

[57] Heuts F., Rottenberg M.E., Salamon D., Rasul E., Adori M., Klein G., Klein E., Nagy N. (2014). T cells modulate Epstein-Barr virus latency phenotypes during infection of humanized mice. J. Virol..

[58] Sato K., Misawa N., Nie C., Satou Y., Iwakiri D., Matsuoka M., Takahashi R., Kuzushima K., Ito M., Takada K. (2011). A novel animal model of Epstein-Barr virus-associated hemophagocytic lymphohistiocytosis in humanized mice. Blood.

[59] Sashihara J., Burbelo P.D., Savoldo B., Pierson T.C., Cohen J.I. (2009). Human antibody titers to Epstein-Barr Virus (EBV) gp350 correlate with neutralization of infectivity better than antibody titers to EBV gp42 using a rapid flow cytometry-based EBV neutralization assay. Virology.

[60] Delecluse H.J., Hilsendegen T., Pich D., Zeidler R., Hammerschmidt W. (1998). Propagation and recovery of intact, infectious Epstein-Barr virus from prokaryotic to human cells. Proc. Natl. Acad. Sci. USA.

[61] Cui X., Cao Z., Wang S., Adler S.P., McVoy M.A., Snapper C.M. (2020). Immunization with Human Cytomegalovirus Core Fusion Machinery and Accessory Envelope Proteins Elicit Strong Synergistic Neutralizing Activities. Vaccines.

[62] Cui X., Cao Z., Sen G., Chattopadhyay G., Fuller D.H., Fuller J.T., Snapper D.M., Snow A.L., Mond J.J., Snapper C.M. (2013). A novel tetrameric gp350 1-470 as a potential Epstein-Barr virus vaccine. Vaccine.

[63] Cui X., Cao Z., Wang S., Flora M., Adler S.P., McVoy M.A., Snapper C.M. (2019). Immunization of Rabbits with Recombinant Human Cytomegalovirus Trimeric versus Monomeric gH/gL Protein Elicits Markedly Higher Titers of Antibody and Neutralization Activity. Int. J. Mol. Sci..

[64] Kimura H., Morita M., Yabuta Y., Kuzushima K., Kato K., Kojima S., Matsuyama T., Morishima T. (1999). Quantitative analysis of Epstein-Barr virus load by using a real-time PCR assay. J. Clin. Microbiol..

[65] Elgui de Oliveira D., Muller-Coan B.G., Pagano J.S. (2016). Viral Carcinogenesis Beyond Malignant Transformation: EBV in the Progression of Human Cancers. Trends Microbiol..

[66] Shannon-Lowe C., Rickinson A. (2019). The Global Landscape of EBV-Associated Tumors. Front. Oncol..

[67] Chen X.Z., Chen H., Castro F.A., Hu J.K., Brenner H. (2015). Epstein-Barr virus infection and gastric cancer: A systematic review. Medicine.

[68] Lee J.H., Kim S.H., Han S.H., An J.S., Lee E.S., Kim Y.S. (2009). Clinicopathological and molecular characteristics of Epstein-Barr virus-associated gastric carcinoma: A meta-analysis. J. Gastroenterol. Hepatol..

[69] Mawson A.R., Majumdar S. (2017). Malaria, Epstein-Barr virus infection and the pathogenesis of Burkitt’s lymphoma. Int. J. Cancer.

[70] Rochford R., Moormann A.M. (2015). Burkitt’s Lymphoma. Curr. Top. Microbiol. Immunol..

[71] Bornkamm G.W. (2009). Epstein-Barr virus and the pathogenesis of Burkitt’s lymphoma: More questions than answers. Int. J. Cancer.

[72] Shanbhag S., Ambinder R.F. (2018). Hodgkin lymphoma: A review and update on recent progress. CA Cancer J. Clin..

[73] Ambinder R.F. (2007). Epstein-barr virus and hodgkin lymphoma. Hematol. Am. Soc. Hematol. Educ. Program.

[74] Weiss L.M., Movahed L.A., Warnke R.A., Sklar J. (1989). Detection of Epstein-Barr viral genomes in Reed-Sternberg cells of Hodgkin’s disease. N. Engl. J. Med..

[75] Anagnostopoulos I., Herbst H., Niedobitek G., Stein H. (1989). Demonstration of monoclonal EBV genomes in Hodgkin’s disease and Ki-1-positive anaplastic large cell lymphoma by combined Southern blot and in situ hybridization. Blood.

[76] Rooney C.M., Smith C.A., Ng C.Y., Loftin S., Li C., Krance R.A., Brenner M.K., Heslop H.E. (1995). Use of gene-modified virus-specific T lymphocytes to control Epstein-Barr-virus-related lymphoproliferation. Lancet.

[77] Rooney C.M., Smith C.A., Ng C.Y., Loftin S.K., Sixbey J.W., Gan Y., Srivastava D.K., Bowman L.C., Krance R.A., Brenner M.K. (1998). Infusion of cytotoxic T cells for the prevention and treatment of Epstein-Barr virus-induced lymphoma in allogeneic transplant recipients. Blood.

[78] Heslop H.E., Ng C.Y., Li C., Smith C.A., Loftin S.K., Krance R.A., Brenner M.K., Rooney C.M. (1996). Long-term restoration of immunity against Epstein-Barr virus infection by adoptive transfer of gene-modified virus-specific T lymphocytes. Nat. Med..

[79] Heslop H.E., Slobod K.S., Pule M.A., Hale G.A., Rousseau A., Smith C.A., Bollard C.M., Liu H., Wu M.F., Rochester R.J. (2010). Long-term outcome of EBV-specific T-cell infusions to prevent or treat EBV-related lymphoproliferative disease in transplant recipients. Blood.

[80] Gottschalk S., Rooney C.M. (2015). Adoptive T-Cell Immunotherapy. Curr. Top. Microbiol. Immunol..

[81] de Martel C., Ferlay J., Franceschi S., Vignat J., Bray F., Forman D., Plummer M. (2012). Global burden of cancers attributable to infections in 2008: A review and synthetic analysis. Lancet Oncol..

[82] Janz A., Oezel M., Kurzeder C., Mautner J., Pich D., Kost M., Hammerschmidt W., Delecluse H.J. (2000). Infectious Epstein-Barr virus lacking major glycoprotein BLLF1 (gp350/220) demonstrates the existence of additional viral ligands. J. Virol..

[83] Draper S.J., Angov E., Horii T., Miller L.H., Srinivasan P., Theisen M., Biswas S. (2015). Recent advances in recombinant protein-based malaria vaccines. Vaccine.

[84] Hjerrild K.A., Jin J., Wright K.E., Brown R.E., Marshall J.M., Labbe G.M., Silk S.E., Cherry C.J., Clemmensen S.B., Jørgensen T. (2016). Production of full-length soluble Plasmodium falciparum RH5 protein vaccine using a Drosophila melanogaster Schneider 2 stable cell line system. Sci. Rep..

[85] Diamond D.J., La Rosa C., Chiuppesi F., Contreras H., Dadwal S., Wussow F., Bautista S., Nakamura R., Zaia J.A. (2018). A fifty-year odyssey: Prospects for a cytomegalovirus vaccine in transplant and congenital infection. Expert Rev. Vaccines.

[86] Cui X., Snapper C.M. (2019). Development of novel vaccines against human cytomegalovirus. Hum. Vaccines Immunother..

[87] Cui X., Cao Z., Wang S., Lee R.B., Wang X., Murata H., Adler S.P., McVoy M.A., Snapper C.M. (2018). Novel trimeric human cytomegalovirus glycoprotein B elicits a high-titer neutralizing antibody response. Vaccine.

[88] Chiuppesi F., Nguyen J., Park S., Contreras H., Kha M., Meng Z., Kaltcheva T., Iniguez A., Martinez J., La Rosa C. (2018). Multiantigenic Modified Vaccinia Virus Ankara Vaccine Vectors To Elicit Potent Humoral and Cellular Immune Reponses against Human Cytomegalovirus in Mice. J. Virol..

[89] McVoy M.A., Lee R., Saccoccio F.M., Hartikka J., Smith L.R., Mahajan R., Wang J.B., Cui X., Adler S.P. (2015). A cytomegalovirus DNA vaccine induces antibodies that block viral entry into fibroblasts and epithelial cells. Vaccine.

[90] Schleiss M.R., Permar S.R., Plotkin S.A. (2017). Progress toward Development of a Vaccine against Congenital Cytomegalovirus Infection. Clin. Vaccine Immunol..

[91] Majji S., Wijayalath W., Shashikumar S., Pow-Sang L., Villasante E., Brumeanu T.D., Casares S. (2016). Differential effect of HLA class-I versus class-II transgenes on human T and B cell reconstitution and function in NRG mice. Sci. Rep..

[92] Majji S., Wijayalath W., Shashikumar S., Brumeanu T.D., Casares S. (2018). Humanized DRAGA mice immunized with Plasmodium falciparum sporozoites and chloroquine elicit protective pre-erythrocytic immunity. Malar. J..

[93] Mendoza M., Ballesteros A., Qiu Q., Pow Sang L., Shashikumar S., Casares S., Brumeanu T.D. (2018). Generation and testing anti-influenza human monoclonal antibodies in a new humanized mouse model (DRAGA: HLA-A2. HLA-DR4. Rag1 KO. IL-2Rgammac KO. NOD). Hum. Vaccines Immunother..

